# Experimental Study on the Impact of Using FRP Sheets on the Axial Compressive Performance of Short-Circular Composite Columns

**DOI:** 10.3390/ma16196373

**Published:** 2023-09-24

**Authors:** Jie Liu, Deliang Ma, Feifei Dong, Zhongxiang Liu

**Affiliations:** 1 College of Civil Engineering, Nanjing Forestry University, Nanjing 210037, China; deliang_ma@outlook.com; 2 National Engineering Research Center of Biomaterials, Nanjing Forestry University, Nanjing 210037, China; feifei_dong@outlook.com; 3 School of Transportation, Southeast University, Nanjing 210096, China; zhongxiang@seu.edu.cn

**Keywords:** FRP, composite column, axial compress, bearing capacity, refinement

## Abstract

This paper conducts an experimental study on the axial compressive performance of FRP-steel-concrete composite columns. Nine short columns were produced and evaluated in the study, comprising of three concrete-filled steel tube reference columns and six FRP-steel-concrete composite columns, respectively denoted as “reference columns” and “composite columns”. Two categories of failure modes, including shear failure and waist drum, were observed from the experiments. The failure mode may trend toward waist drum from shear failure as more FRP layers were used. The number of FRP layers had a direct effect on the level of compressive strength attained, with a greater number of layers resulting in a greater increase in compressive strength. Moreover, a greater tensile strength and higher elastic modulus of CFRP sheets are more effective at improving the compressive stiffness of the columns. Finally, a four-stage confinement mechanism for FRP-wrapped steel tube concrete composite columns is proposed and discussed, through which the damage mechanisms of the composite structures are more rationally characterized.

## 1. Introduction

Concrete-filled steel tube (CFST) columns are widely utilized in civil engineering structures due to their significant enhancement of the performance of inner concrete facilitated by steel tube confinement [1,2,3,4,5]. However, the outer steel tubes of these columns are susceptible to experiencing local buckling and erosion damage in service [6,7,8,9,10].

In order to improve the pressure-bearing performance of CFSTs and their ability to resist environmental erosion, high-performance or functional materials have been adopted to wrap and reinforce CFSTs. The most typical method is the augmentation method of wrapping FRP layers [11,12,13] and using shape memory alloy (SMA) [14,15]. These reinforcement techniques all make use of the superior mechanical properties of external materials, such as the light weight and high strength of FRP materials [16,17,18,19,20], as well as the high ductility and good energy dissipation capacity of SMA [15].

One pioneer study on the axial bearing performance of FRP-wrapped CFSTs was written by Tao et al. [21]. In their study, both rectangular and circular composite specimens retrofitted with CFRP were tested. The experimental findings unequivocally demonstrate that the load-bearing strength of retrofitted circular column specimens was significantly improved by CFRP composites, whereas only limited improvement was observed in the case of rectangular columns. After that, Liu et al. [22] carried out an experimental investigation of 11 short column specimens under axial compressing loads and proposed corresponding formulas for calculating the bearing strength of the FRP-CFST columns. Hu et al. [23] carried out an experimental study to investigate the behavior of FRP-confined CFSTs under axial compression. The findings of the study revealed that the application of FRP confinement can result in a noteworthy enhancement of both the carrying strength and ductility of CFSTs. Na et al. [24] subsequently conducted an inquiry into the slenderness effects of CFTs confined with CFRP. Their study demonstrated that the composite wraps provided effective confinement to the inner CFST, thus enhancing the performance of the CFST columns. However, it was observed that the reinforcing effect of the CFRP wrapping layers decreased with an increasing slenderness ratio. More recently, Zhang et al. [25] proposed a stress–strain analysis model of FRP-confined CFSTs under axial compression, based on an experimental investigation. Wang et al. [7] explored the axial compressive performance and confinement mechanisms of circular FRP-steel tube concrete stub columns and determined that the capacity of the CFST specimens was augmented by reducing the steel tube’s diameter-to-thickness ratio. In 2022, Onyelowe et al. [26,27] investigated the compressive strength of CFRP-wrapped concrete columns using AI (artificial intelligence) techniques and concluded that the strength of wrapped concrete columns depends on the FRP thickness and tensile strength.

**Figure 1:**
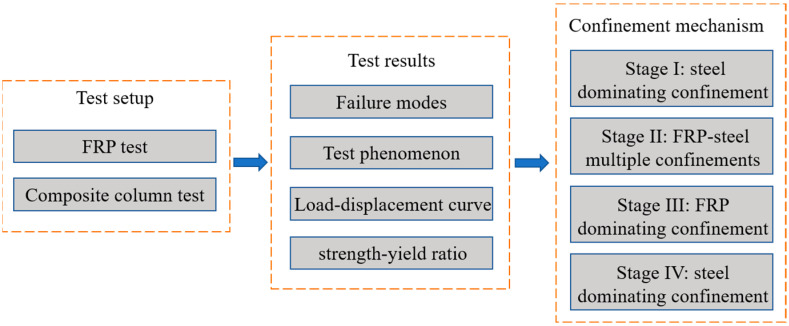
Research methodology of this study.

Although previous studies have addressed the bearing strength of FRP-wrapped CFST columns, few studies have investigated the complex load-bearing effects of composite columns—particularly the failure mechanism of composite columns under axial forces, and the effect of different FRP materials (and thicknesses) on load-bearing capacity. This paper examines the characteristics and mechanism of compression failure in CFST columns strengthened with wraps composed of two different FRP materials with varying thicknesses. The failure mechanism of composite columns will be investigated to gain a deeper understanding of the factors affecting such mechanisms. Finally, based on the experimental results, a four-stage failure mechanism of an FRP-steel-concrete column under axial compress is proposed. The research methodology of this study is illustrated in Figure 1.

## 2. Experimental Program

### 2.1. Specimen Configurations and Material Properties

**Figure 2:**
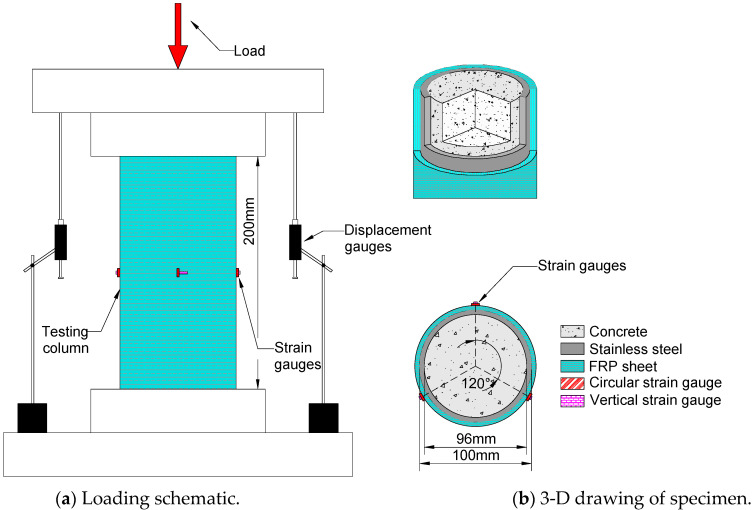
Diagram of the composite column (unit: mm).

In order to study the effect of FRP wraps on the compressive performance of CFST, 9 short columns were fabricated, including 3 CFST columns (referred to as “reference columns”) and 6 FRP-steel-concrete composite columns (FRP sheets wrapped outside the reference column, referred to as “composite columns”) were manufactured. Detailed information about the columns is listed in Table 1. The CFST specimens were all poured into hollow steel tubes, and the outer diameter *D* and thickness of the steel tubes were 100 mm and 2 mm, respectively. The composite column and the test setup are plotted in Figure 2. The thickness of a single layer of FRP was 0.167 mm. For each composite column, FRP was affixed onto the outer surface of the steel tube using steel glue, and the FRP interface of adjacent layers was also bonded together via steel glue. The tensile strength of the steel glue material was 44.2 MPa, which was provided by the manufacturer.

For the convenience of expression, each specimen was named separately. As in Table 1, the 3 reference columns are donated as N1, N2, and N3. The 6 composite column specimens are donated as CC1, CC2, CC3, CB1, CB2, and CB3. The adhesive was epoxy resin-type steel glue with a tensile strength of 44.2 MPa.

**Figure 3:**
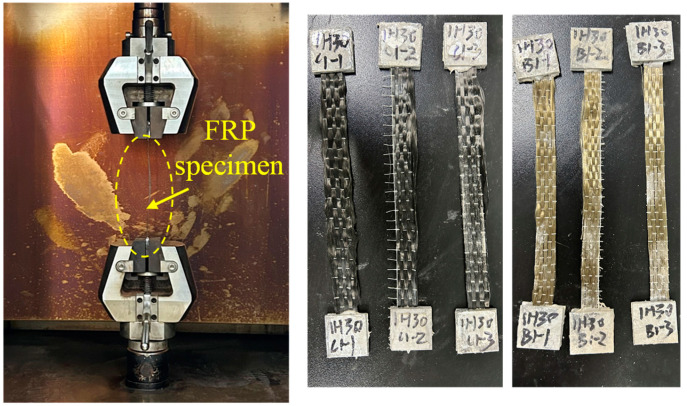
Loading and failure diagrams of FRP specimens.

The FRP wrapping utilized in this study was sourced from Jiangsu Green Material Valley New Material Technology Development Co., Ltd. Both CFRP (Carbon Fiber Reinforced Polymer) and BFRP (Basalt Fiber Reinforced Polymer) composites were employed in the sample specimens. The Young’s moduli of CFRP and BFRP were 1.67 × 10^5^ and 9.24 × 10^4^ MPa respectively, as provided by the manufacturer. The steel tubes were made of 06Cr19Ni10 stainless steel (i.e., SUS 304 stainless steel in the US and Japan), and the tensile strength of the steel material was 550 MPa. The average yield strength and Young’s modulus were measured as 207 MPa and 198 GPa, respectively, by the manufacturer. The concrete inner core was C60 grade. The compressive strength of the concrete cube was measured via a cube test block with a side length of 150 mm, according to the “Concrete Strength Inspection and Evaluation Standard” (GB/T50107-2019). The average compressive strength of the cubes was measured to be 61.4 MPa. To precisely determine the material properties of the FRP sheets, a tensile test of six FRP specimens (including three CFRP and three BFRP) was fabricated. The sizes of the FRP specimens were designed as 100 mm (length) × 10 mm (width) × 0.167 mm (thickness), respectively, according to the recommendation of “Carbon fiber sheets for structural reinforcement and repair” (JG/T 167-2016). The loading and failure diagram of the test for FRP sheets is depicted in Figure 3. The average tensile strengths of the CFRP and BFRP sheets were measured as 4101.0 and 2005.1 MPa, respectively. The material properties of FRP and steel are listed in Table 2.

### 2.2. Test Setup

**Figure 4:**
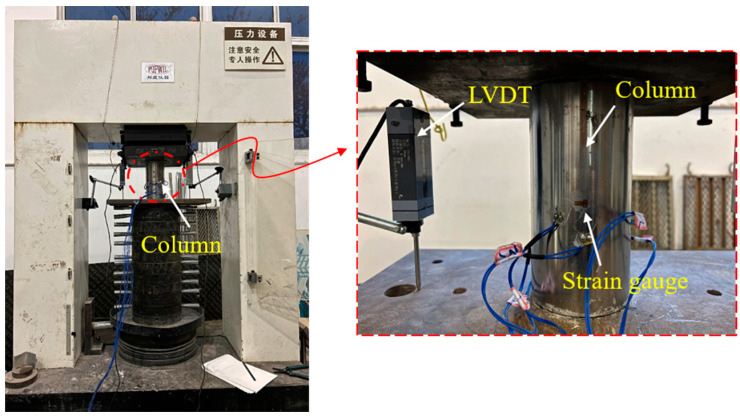
Test setup and instrumentation of the composite columns.

The axial compression loading test was conducted utilizing the Bonwe 3000 kN high-rigidity geotechnical concrete testing machine, and the test setup is depicted in Figure 4. The loading mechanism was executed in the following manner:

Preloading: The column specimen’s axis was positioned at the central axis of the testing machine to ensure that the specimen bore the force on the axis and remained within the range of the elastic loading stage of the specimen. The loading process was initiated by preloading the specimen up to approximately 10% of the estimated ultimate load at a loading rate of 2 mm/min, and the specimen was subjected to preloading three times. This ensured that the instrument worked correctly and the influence of the gap between the test specimen and the tool was eliminated.

Formal loading: Each specimen underwent monotonic axial compression testing, with the complete loading process utilizing a program for automatic control of axial displacement. The load was applied with the displacement-control mode at a rate of 2 mm/min. The specimen’s load increased until it was damaged (FRP fracture, steel pipe yield) or the loading was stopped when the load declined to 80% of the peak load. The specimen was unloaded afterward.

The measurement items for the columns’ monotonic axial compression test primarily entailed information on the vertical load, vertical compression, and horizontal and vertical strain in the middle plane of the specimen. The vertical load was evaluated using the built-in dynamometer of the press, and the vertical compression was calculated utilizing the vertical relative displacement of the loading plate at both ends of the specimen during the loading process as measured by the displacement gauges, as demonstrated in Figure 1 and Figure 3. The horizontal and vertical strain gauges in the middle of the specimen measured the horizontal and vertical strain, respectively. The strain gauges were arranged at the middle height of the specimen in the trisecting circle, with three pairs of vertically arranged strain gauges employed to record the lateral strain gauge distribution. The strain gauge model BX120-15AA, with a grid length of 15 mm × 3 mm and a resistance value of 120 ± 0.19 Ω, was adopted. During the loading process, the load and displacement were collected by the data acquisition system TDS630 automatically at a sampling frequency of 50 Hz.

### 2.3. Mechanical Conditions of Composite Columns

Considering that the FRP-steel-concrete composite short column is a composite structural system, the load-bearing state of the concrete core is the key factor affecting the bearing strength of the composite columns. The mechanical behavior of the concrete inside is then analyzed. The inner concrete of the columns is in a typical triaxial stress condition. Based on the discussion of Liu et al. [22], the axial compressive strength of the inner concrete is in a linear relationship with the lateral confining pressure as follows:

(1) $$f_{cc}=f_{c}+kp,$$

where *f_cc_* and *f_c_* are the axial compressive strength of the inner concrete with and without confinement, respectively, *p* denotes the magnitude of the lateral confining pressure, and *k* denotes the strength-increasing coefficient of the inner concrete. From this formula, it can be seen that the stronger the confinement effect of the outer wrapped FRP and the steel pipe, the higher the bearing strength of the inner core concrete column.

**Figure 5:**
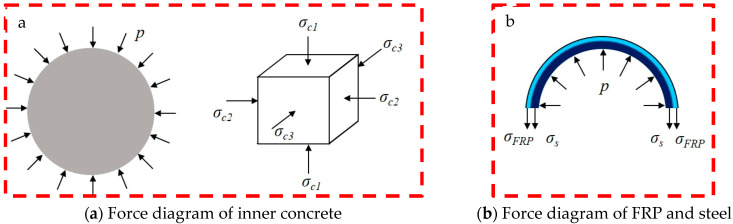
Force diagram of inner concrete and FRP-steel layer.

Figure 5 plots the force of the inner concrete and FRP-steel layer. *σ_c_*_1_ is the axial press of the concrete. *σ_c_*_2_ and *σ_c_*_3_ are the principal stresses in the other two directions; they have the following relation:

(2) $$\sigma_{c1}=f_{cc}=f_{c}+kp,$$

(3) $$\sigma_{c2}=\sigma_{c2}=p,$$

Based on the force diagram of the FRP-steel layer in Figure 4, the lateral confining pressure *p* can be adopted as follows:

(4) $$p=(\sigma_{s}\times 2t_{s}+\sigma_{FRP}\times 2t_{FRP})/D,$$

where *σ_s_* and *σ_FRP_* denote the transverse stresses of the steel pipe and FRP sheets, respectively; *t_s_* and *t_FRP_* denote the thicknesses of the steel pipe and FRP sheets, respectively; and *D* denotes the diameter of the inner concrete column. By combining Equations (1) and (4), the final expression can be obtained:

(5) $$f_{cc}=f_{c}+k(\sigma_{s}\times 2t_{s}+\sigma_{FRP}\times 2t_{FRP})/D,$$

Equation (5) shows that for steel-concrete columns wrapped in FRP, if the circumferential confinement stress or the thickness of the confining circle of the outer steel pipe and FRP layer increases, the compressive strength of the composite column will be effectively improved accordingly.

For the CFST column, the confinement effect of the steel on the inner concrete can be expressed as the following:

(6) $$\xi_{ss}=\frac{A_{ss}\sigma_{0.2}}{A_{c}\times 0.833\times f_{cu}}$$

where *A_ss_* is the cross-sectional area of the steel tube; $\sigma_{0.2}$ is the 0.2% proof stress of the stainless steel; *A_c_* is the cross-sectional area of the concrete; and *f_cu_* is the strength of the concrete cube.

Han et al. [28] proposed the equation for bearing capacities of circular CFST specimens, which is shown as the following:

(7) $$N_{u}=(1+b\xi_{ss})f_{c}^{\prime }A_{c}$$

where *N_u_* is the bearing capacity of the CFST column, $f_{c}^{\prime }$ is the strength of a standard concrete cylinder, and *b* is the influence parameter of steel refinement.

## 3. Experimental Results

### 3.1. Failure Modes

**Figure 6:**
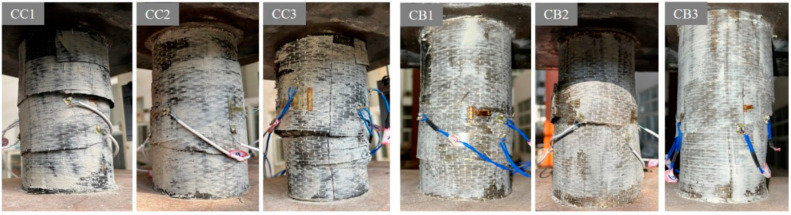
Pictures of the composite columns after destruction.

**Figure 7:**
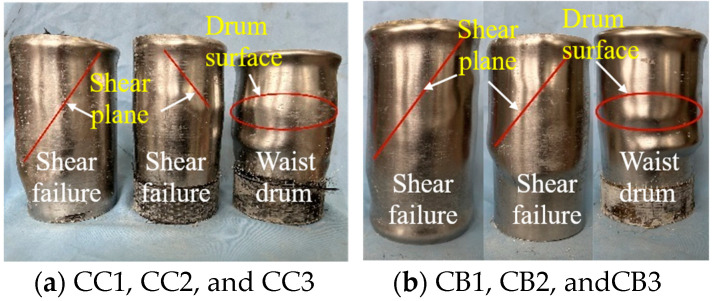
Two failure modes of composite specimens.

After testing the specimens, the damaged specimens are shown in Figure 6. After continuous axial compression, the FRP layer on the surface of the specimen was fractured and degummed. As shown in Figure 7, the FRP constraining layer of the composite columns was peeled off to study the failure mode of the composite columns. Based on the axial compression tests, two failure modes of waist drum and shear failure were observed. Typical shear failure was found in specimens CC1, CC2, CB1, and CB2, whereas waist drum failure was found in CC3 and CB3, indicating that as the number of FRP layers increased, the failure mode of the composite columns may trend to be waist drum from shear failure. The destruction mechanism can be described as follows: for the reference columns, as the concrete compressed, due to the limited restraint capacity of the outer wrapping of the steel tube, the shear failure of the inner core concrete column occurred at a lower load. However, for composite columns, the expansion of internal concrete is restrained by FRP-steel composite confinement, leading to a significant enhancement of the compressive performance of internal concrete. Due to the strong external constraints of the double layers of FRP and steel pipe, the inner core concrete column was always in a state of obvious triaxial compression. The failure of the concrete column is more inclined to material destruction, resulting in the waist drum.

### 3.2. Test Phenomenon

**Figure 8:**
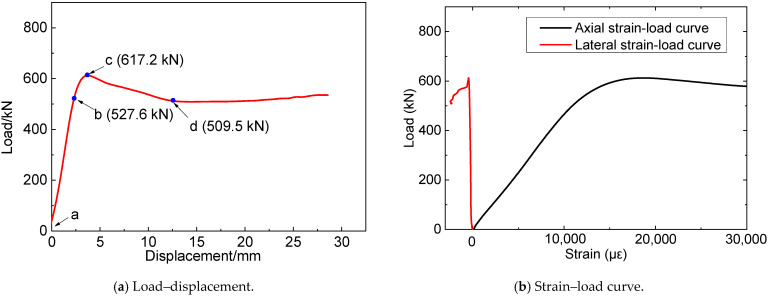
Figure of the load–displacement and strain–load curve of N1.

**Figure 9:**
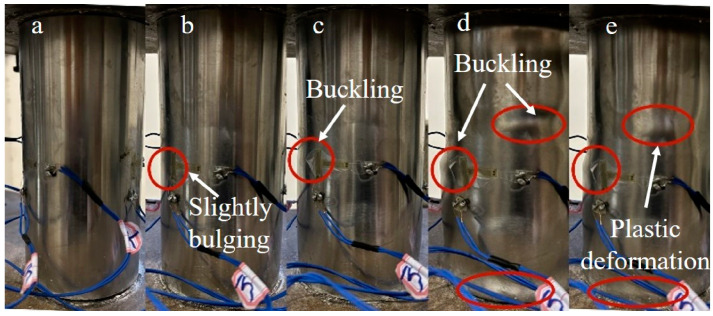
Figure of failure process for N1 under axial compression. (**a**) The initial loading stage; (**b**) The stage of yielding of steel tube; (**c**) The stage when the axial compression load reaches its maximum value; (**d**) The stage when the axial compressive load reaches the lowest value after the peak value; (**e**) Continuous compression stage after buckling.

Figure 8 plots the load–displacement relationship of specimen N1, within which “a” represents the loading moment, “b” indicates the initiation of the nonlinear stage, “c” denotes the bearing capacity (peak), and “d” signifies the residual load after bearing capacity (peak). Based on the experimentally observed phenomena, during the initial loading stage, the load–displacement curve of the specimen exhibited a nearly linear increase, with no apparent surface damages observed, as shown in Figure 9a. Upon reaching the yield load point of the steel tube, a slight bulging was noticed on its surface, as depicted in Figure 9b. Eventually, as the load approached the ultimate bearing capacity, clear instances of buckling deformation occurred on the surface of the steel tube, as illustrated in Figure 9c. Subsequently, the bearing capacity of the specimens gradually decreased. Upon the load reaching its lowest point, the original steel tube’s buckling deformation heightened, with novel buckling deformation emerging, as shown in Figure 9d. As the specimen endured continuous compression, its lateral deformation rose persistently, culminating in the formation of several distinct local plastic deformations, as depicted in Figure 9e.

**Figure 10:**
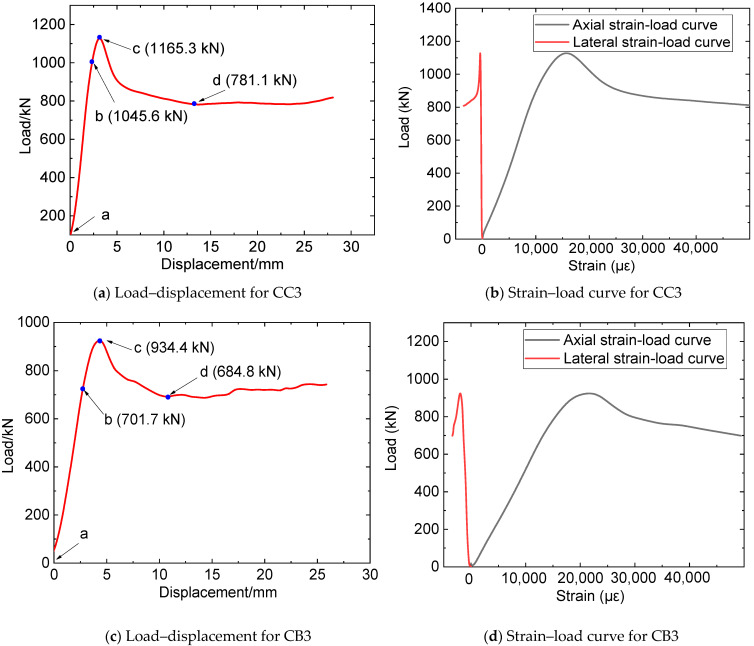
Figures of the load–displacement and strain–load curves for CC3 and CB3.

**Figure 11:**
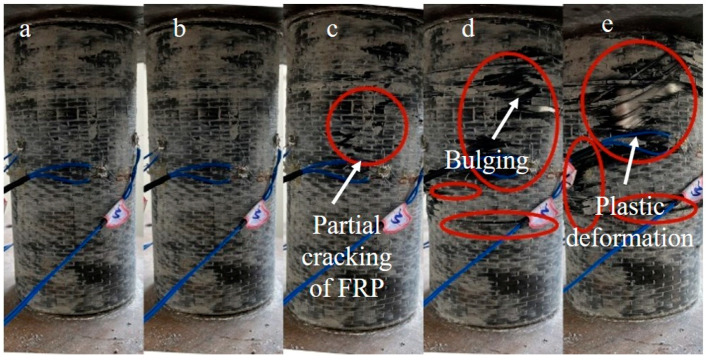
Failure process of the CC3 specimen under axial compression. (**a**) The initial loading stage; (**b**) The stage of slight glue cracking; (**c**) The stage when the axial compression load reaches its maximum value; (**d**) The stage when the axial compressive load reaches the lowest value after the peak value; (**e**) Continuous compression stage after buckling.

Similar testing phenomena were observed in the six composite columns. Take CC3 as an example. The load–displacement curve is plotted in Figure 10, where “*a*”~“*b*” have identical meanings to those of Figure 8. Similar to the reference columns, during the initial loading stage (Figure 10a), the load–displacement curve of the composite columns increased approximately linearly, with no discernable damage observed on the specimen’s surface. As the loads increased, a slight glue cracking sound was heard, with no obvious change observed, as depicted in Figure 11b. When the loads reached the specimen’s ultimate load (Figure 11c), evident glue cracking sounds were observed, with localized cracks appearing on the FRP surface, indicating that the cemented and reinforced outer layer had begun to disintegrate and fail in large quantities. Subsequently, the FRP cracks expanded rapidly, with the surface of the steel pipe bulging slightly and the bearing load beginning to gradually decline. The load continued to decline to the lowest point; at this point, there were hardly any new cracking positions on the FRP surface, as shown in Figure 11d. Ultimately, several distinct local plastic deformations were formed at the FRP fracture, as shown in Figure 11e. The load–displacement curve of specimen CB3 was plotted in Figure 10b, and a similar phenomenon to CC3 was observed.

#### 3.2.1. Load–Displacement Relationship

**Figure 12:**
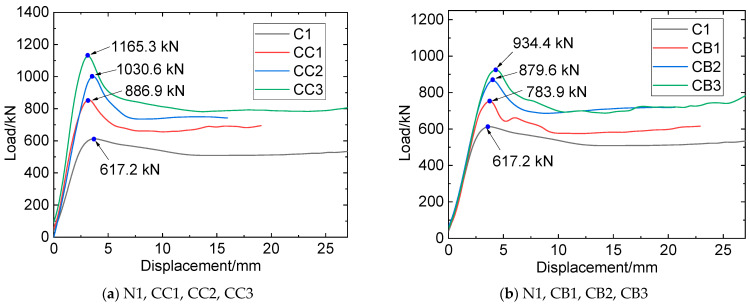
Load–displacement relationship.

The experimental findings revealed that the load–displacement curves of both the reference and composite specimens can be discerned into four distinct stages: linear elastic, elastic–plastic, descending, and steady. Figure 12 presents a comparative analysis of the load–displacement relationships for the N1, CC1, CC2, and CC3 specimens.

From Figure 12, it can be seen that, at the initial stage, as the compression displacement increases, the load of the N1 specimen also linearly increases, indicating that the specimen is in the elastic stage. With the further increase of compression displacement (segment b–c), the load–displacement curve shows a quadratic feature, indicating that the specimen is in the elastic–plastic stage. After a short elastic–plastic stage, the load of the specimen reaches its peak at point B, where the bearing capacity is 617.2 kN. Thereafter, as the displacement continues to increase, steel tube buckling deformation increases steeply, and the load begins to decrease. With the continuous expansion of concrete, steel material continues to deform and enters the hardening stage, resulting in a slight increase in confining pressure on the concrete and entering the steady stage.

Compared with the N1 specimen, CC1, CC2, and CC3 show longer elastic–plastic stages and larger peak loads due to the constraint effect of CFRP on the steel-concrete column, with the peak loads (bearing capacities) of 886.9 kN, 1030.6 kN, and 1165.3 kN, respectively. When the compressing load reaches the peak, FRP begins to rupture, and the load–displacement curve enters the descending stage. When the load reaches the lowest point, FRP fractures extensively, and thereafter, the specimen continues to be compressed and the steel material remains in the yield stage. When entering the steady state, the load of the specimen is in the micro-fluctuation stage. Comparing the curves of the CC1, CC2, and CC3 specimens, it can be concluded that, with the increase in the number of CFRP layers, the slope of the load–displacement curve in the elastic stage gradually increases, and the starting point of the elastic–plastic stage increases while the corresponding displacement decreases.

The load–displacement curves of the CB1, CB2, and CB3 specimens are illustrated in Figure 11b. They show similar characteristics to the CC1, CC2, and CC3 specimens. It is noteworthy that, compared with specimens reinforced by CFRP, the peak loads of specimens reinforced by BFRP are relatively small, with the compressing capacities of 783.9 kN (CB1), 879.6 kN (CB2), and 934.4 kN (CB3).

In summary, during the linear elastic stage, a similar phenomenon of the load–displacement curve of CFRP- or BFRP-constrained specimens could be observed. This may be attributed to the small expansion deformation of the concrete at this stage, resulting in limited compression on the steel pipe. Consequently, the steel tube remains at the linear elastic stage with the FRP and the steel tube deforming in coordination, resulting in small forces on the FRP. As the specimen was continuously compressed and entered the elastic–plastic stage, the concrete underwent significant expansion deformation, the steel tube endured large stress and produced plastic deformation, and the restraining effect of the FRP became increasingly significant. The ultimate bearing capacity of the steel tube enhanced by CFRP is more significant than that by BFRP, which can be attributed to the bigger elastic modulus and higher tensile strength of CFRP. In addition, more FRP wrapping layers cause an increase in the bearing capacity of the composite columns, indicating that wrapping FRP sheet is an effective method to improve the bearing performance of CFST columns.

#### 3.2.2. Mechanical Characteristics Analysis

Based on the results of Figure 12, the bearing capacities of N1, CC1, CC2, and CC3 were 617.2 kN, 886.9 kN, 1030.6 kN, and 1165.3 kN, respectively. Compared with the N1 specimen, the increasing ratio of the bearing capacities of CC1, CC2, and CC3 was calculated as 43.7%, 66.8%, and 88.6%, respectively. Those of CB1, CB2, and CB3 were 26.9%, 42.3%, and 51.1% compared to the N1 specimen. The strength-yield ratio is defined as the ratio of the ultimate bearing strength to the yield strength of the tested specimens, as Equation (8) shows.

(8) $$R_{sy}=\frac{f_{y}}{f_{c}},$$

where *R_sy_* is the strength-yield ratio, and *f_y_* and *f_c_* are the yield strength and ultimate strength of the columns, respectively.

**Figure 13:**
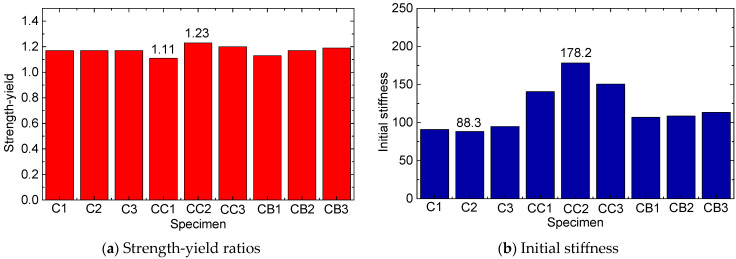
Strength-yield ratios and initial stiffnesses of the nine columns.

The strength-yield ratios of all nine specimens were calculated as 1.17 (N1), 1.17 (N2), 1.17 (N3), 1.11 (CC1), 1.23 (CC2), 1.20 (CC3), 1.13 (CB1), 1.17 (CB2), and 1.19 (CB3), as presented in Figure 13a and Table 2. The strength-yield ratios of all nine specimens ranged between 1.11 and 1.20, indicating that the wrapped FRP sheets have a negligible effect on the strength-yield ratio of the composite specimens. Further study was conducted to analyze the influence of the FRP layer on the compressive stiffness of the composite column. This was performed by investigating the initial stiffness, which is defined as the linear stiffness of the specimen during the initial linear elastic compression process (i.e., section a~b in the load–displacement curve). The calculated outcomes are presented in Figure 13b and Table 2. The results indicate that the initial stiffness of the composite specimens ranges between 100 kN/mm and 180 kN/mm, reaching a maximum of 178.2 kN/mm for CC2 and a minimum of 106.9 kN/mm for CB1. The average initial stiffness for the N1, N2, and N3 specimens was recorded at 91.2 kN/mm. These findings show that the outer FRP sheets can notably enhance the initial stiffness of the CFST specimens. Notably, the initial stiffness values for the CFRP-reinforced specimens are significantly higher than those of the BFRP-reinforced specimens. This supports the inference that the higher elastic modulus of the CFRP sheets is more effective in improving the compressive stiffness of the column specimens. Table 3 illustrates a summary of the characteristic parameters for all the tested specimens.

### 3.3. Confinement Mechanism of Composite Columns

**Figure 14:**
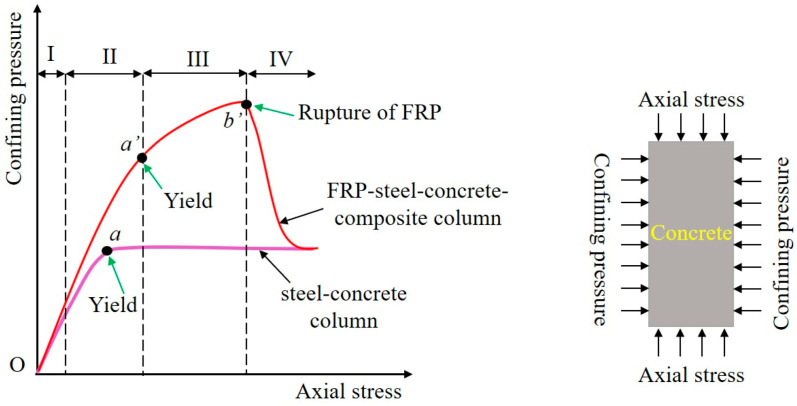
Confinement mechanism of the composite column and reference column.

The aforementioned loading test results of composite columns demonstrate that the outer coating of FRP will change the load-bearing failure mechanism of the short column and enhance its bearing capacity. The change in this load-bearing mechanism is embodied in the confinement mechanism of the FRP layer, which is discussed in this section, as shown in Figure 14 (red curve). The confinement mechanism being proposed can be classified into four distinct stages.

(1) Stage I: steel dominating confinement

In the initial loading stage, the axial compression effect is minimal, and the lateral deformation of the inner concrete results primarily from Poisson’s effect and the emergence of microcracks. As activation is insufficient at this stage, only a slight rise in total confining pressure is observed. The bulk of the confinement occurs as a result of the steel pipe, with the FRP being inconsequential due to inadequate activation. Throughout this stage, the axial stress–strain reaction of the internal concrete registers on the first ascending branch.

(2) Stage II: FRP-steel multiple confinements

With an increase in axial strain, gradual radial dilation is observed in the internal concrete, accompanied by the initiation and propagation of minor cracks. Consequently, the steel and FRP confinement forces become fully activated, inducing a much faster acceleration in their increase rate compared to that observed earlier. This surge in forces leads to the swift development of total confining pressure. To accurately determine the boundary of this phase, this deviation must be captured. Additionally, the steel tube exhibits yielding at the end of the second stage, which can be defined as a critical boundary line. The duration of this stage hinges on the yield strength and yield strain of the steel tube, with higher values of these parameters extending the duration of the second stage proportionately.

(3) Stage III: FRP dominating confinement.

As the bearing pressure continues to increase, the FRP confining pressure increases gradually owing to the progressive radial expansion of interstitial concrete, which are accompanied by the appearance of numerous macroscopic cracks. Conversely, the steel confining pressure stays mostly stable after yielding. As a consequence, the total confining pressure increases at a slower pace. ‘Stage III’ culminates when the internal concrete reaches its ultimate axial strain (*ε_u_*), leading to the rupture of the FRP hoop (represented by point *b*′ in Figure 13). The axial stress–strain response of the internal concrete during this stage aligns roughly with the second ascending branch. Hence, the mechanical characteristics of the FRP layers play a critical role in the confinement effect during ‘Stages II and III,’ with stronger FRP layers being most effective in enhancing the ultimate load-bearing capacity of the specimen.

(4) Stage IV: steel dominating confinement

Following the third stage, the FRP hoop layers are pulled off while the steel pipe remains in a plastic stress state following yielding. During the fourth stage, the confinement pressure is primarily borne by the steel pipe, resulting in the external confinement force being returned to the confining effect of the steel pipe in the concrete inner core. The confinement mechanism during this stage was similar to that observed in a pure steel tube concrete column (i.e., the reference column). As the compression continued, the final failure of the specimen is reflected in the crushing of the concrete core.

## 4. Conclusions

Drawing on experimental research, this study investigated the bearing mechanism and damage process of short columns, including pure steel tube concrete columns and FRP-wrapped steel tube concrete composite columns. The failure process, failure mode, load–displacement relationship, strength-yield ratio, and initial stiffness were discussed. The following conclusions can be drawn:

(1) Two failure modes, shear failure and waist drum, were observed during testing. For FRP-wrapped steel tube concrete composite columns, the failure mode may trend toward waist drum from shear failure as more FRP layers are used. Due to the strong external combining constraints of the FRP and steel tube, the inner core concrete column is always in a state of obvious triaxial compression. Consequently, the failure of the concrete column is more inclined to material destruction, resulting in the waist drum failure mode.

(2) When compared with the pure steel tube concrete column, the CFRP- and BFRP-wrapped columns exhibit larger peak loads, indicating that the effective use of FRP wraps can significantly increase the compressive strength of steel-concrete columns. Furthermore, the number of FRP layers has a direct impact on the level of compressive strength attained, with more layers resulting in a greater increase in the compressive strength.

(3) The incorporation of wrapped FRP sheets has no direct effect on the strength-yield ratio of the composite specimens. However, the initial stiffnesses (the ratio of the load to compressive displacement during the initial stage) of CFRP-reinforced specimens are significantly greater than those reinforced with BFRP. This discrepancy indicates that the higher elastic modulus and greater tensile capacity of CFRP sheets are more effective at improving the compressive stiffness of columns.

(4) This research proposed a four-stage confinement mechanism for FRP-wrapped steel tube concrete composite columns, comprising the following stages: Stage I—steel dominating confinement, Stage II—multiple confinements by both FRP and steel, Stage III—FRP dominating confinement, and Stage IV—steel dominating confinement. The confinement effect during stages II and III is primarily influenced by the mechanical properties of the FRP layer, with higher-strength FRP layers being the most effective at improving the ultimate bearing strength of the specimens.

## Figures and Tables

**Table 1:** Information about the columns.

Category	Specimen No.	FRP Category	Number of FRP Layer	The Total Thickness of FRP Layer/mm
Reference column	N1	N/A	0	0
	N2	N/A	0	0
	N3	N/A	0	0
composite column	CC1	CFRP	1	0.167
	CC2	CFRP	2	0.334
	CC3	CFRP	3	0.501
	CB1	BFRP	1	0.167
	CB2	BFRP	2	0.334
	CB3	BFRP	3	0.501

**Table 2:** Material properties of FRP and steel.

	Young’s Moduli/MPa	Tensile Strength/MPa
CFRP	1.67 × 10^5^	4101.0 (on average)
BFRP	9.24 × 10^4^	2005.1 (on average)
Steel	1.98 × 10^5^	550

**Table 3:** Tested results of the columns.

Specimen No.	Extreme Load/kN	Yield Load/kN	Strength-Yield, μ	Initial Stiffness/(kN/mm)
N1	617.2	528.8	1.17	90.9
N2	617.8	529.3	1.17	88.3
N3	617.1	528.2	1.17	94.8
CC1	886.9	800.1	1.11	140.6
CC2	1030.6	839.6	1.23	178.2
CC3	1165.3	974.7	1.20	150.6
CB1	783.9	691.8	1.13	106.9
CB2	879.6	748.9	1.17	108.6
CB3	934.4	788.3	1.19	113.4

## Data Availability

Data will be made available on request.
